# Patient Partner Perspectives: The Experience of Participating in a Co-Designed Virtual Reality Project

**DOI:** 10.1177/23743735241302932

**Published:** 2024-12-12

**Authors:** Heather Thomson, Lisa Di Prospero, Sarah Xiao, Tamara Harth, Laurie Legere, Laura Rashleigh, Maria Parzanese

**Affiliations:** 1 70379 Lawrence Bloomberg Faculty of Nursing, University of Toronto, Toronto, ON, Canada; 2 Practice-Based Research and Innovation, 71545 Sunnybrook Health Sciences Centre, Toronto, ON, Canada; 3 Department of Radiation Oncology, Temerty Faculty of Medicine, University of Toronto, Toronto, ON, Canada; 4 The Nethersole School of Nursing, Faculty of Medicine, The Chinese University of Hong Kong, Shatin, Hong Kong

**Keywords:** patient engagement, co-design, patient partners

## Abstract

Patient partners (PP) are well positioned to make meaningful contributions to healthcare through their lived experiences and personal narratives. However, researchers must ensure that patients are engaged authentically and collaboratively in knowledge generation. As part of a larger project, 4 PP were engaged in the co-design of a virtual reality video aimed at promoting an understanding of patients’ lived experience with COVID-19 during the initial phase of the pandemic. This paper reports on findings from follow-up evaluation interviews with PP about their experiences participating in this project. Thematic analysis of interview transcripts resulted in 2 major themes as well as facilitators and barriers to PP engagement. Primary reasons to participate in the project were to contribute and give back to the community and make a difference for patients impacted by COVID-19. Engagement resulted in positive experiences and impacts for PP. Facilitators to engagement included feeling heard, being valued, and treated with respect. Barriers included length of time required to complete the project as well as PP health status.

## Key Points

1. Patient partners are well positioned to contribute to the design, implementation, and evaluation of interventions, programs, and services within healthcare.
2. Patient partners chose to participate in a co-design project to contribute and give back to the medical research community and make a difference for patients affected by COVID-19 at the onset of the pandemic. Engagement resulted in positive experiences and impacts for patient partners.
3. Facilitators to engagement included feeling heard, being valued and treated with respect. Barriers included the length of time required to complete the project in addition to patient partner health status.
4. Further research regarding effective engagement and appropriate compensation of patient partners is required.

## Introduction

Patient (patients, family members, caregivers) engagement within the healthcare system is recognized as being fundamental to quality and patient-centered care.^1,2^ Patients have participated in care design and delivery, quality improvement, and education, influencing policy and governance, as well as research.^3-8^ Through varying degrees of engagement, patients have been described as stakeholders, advisors, partners, collaborators, and co-investigators.^3,8,9^ A common element of patient engagement is recognition that they leverage their past and/or current experiences with the healthcare system.^3^ Specifically, patients’ lived experience makes them well positioned to meaningfully participate and contribute as collaborative partners in research, evaluation, and quality improvement activities.

The level and extent of patient partner engagement is influenced by research team goals, patient preferences, and funding opportunities. Theoretical models and frameworks exist to describe and guide patient engagement efforts across the healthcare system.^7,8,10-12^ Co-design is one form of active engagement where patients are involved in designing, planning, implementing, and evaluating interventions, programs, and services.^13^

This paper reports findings from interviews conducted with patient partners (PP) in follow-up to a research project where they were engaged in co-design and pilot testing of a virtual reality (VR) video aimed at promoting empathy among clinicians toward patients experiencing COVID-19^14^ PP were invited to share their experiences to improve our understanding of engaging patients in research, as well as barriers and facilitators to engagement.

## Methods

Four PP with lived experience of COVID-19 were recruited early in the pandemic to participate in the co-design and pilot testing of an educational VR video, along with 7 other interprofessional team members inclusive of nursing and radiation therapy. PP contributed to the development, pilot testing, and dissemination of the VR video by sharing their stories, participating in the development of the video script, post-production editing, and interpretation and dissemination of video pilot testing data (described in more detail in another publication^14^). The initial aim was to recruit up to 10 PP, however, only 4 PP responded to hospital ads and social media posts, possibly due to the timing of recruitment which took place during the summer of 2020, following the first wave of the pandemic. All 4 PP met the criteria of having lived experience and consented to participate as members of the project team. Upon completion of the project, all 4 PP consented to participate in follow-up interviews exploring their experience of being engaged in the project.

A qualitative descriptive design was used. Six semi-structured interview questions were developed by the core study team as part of the original project protocol (Table 1). To promote anonymity, interviews were conducted by a research assistant external to the team and not known to PP.^5,9^ Interviews were conducted virtually using Zoom, audio recorded, and transcribed verbatim. Data were analyzed using inductive thematic analysis using Braun and Clarke's 6 phases.^15^ Transcripts were manually coded independently by 3 researchers who then met to discuss, refine, and define emergent themes.

**Table 1.** Patient Partner Interview Questions.

1. What motivated you to participate in this project? 2. Tell us about your experience participating in this project. How did it impact you? 3. Did anything surprise you about this experience? 4. Were there any benefits to participating? If yes, please describe. 5. Were there any drawbacks to participating? If yes, please describe. 6. Is there anything else that you would like to share?

## Results

Two primary themes were articulated: (1) contributing and giving back and (2) positive experiences and impact, which included 2 subthemes of connecting through a shared experience and feeling validated and empowered. Facilitators, drawbacks, and barriers to engagement were also identified (Table 2).

**Table 2.** Quotes to Illustrate Identified Themes and Subthemes.

Theme	Illustrative quotes
Contributing and Giving Back	“So at the time, I was really just interested in helping out in any way I could. It was early days in the pandemic and I just thought, listen, if I can get some help myself or some information myself and also help ...somebody else in this process, and that's what I would do.” (Participant #1) “I was motivated to participate. If there was any way I could contribute to other COVID patients having a better experience than I did.” (Participant #3) “My experience with getting diagnosed to my follow ups and anyone just believing me in general was so hard that I thought if I can contribute any kind of perspective on how a patient may be feeling, what a patient may need and how we could hopefully change the way that professionals see patients, that I would want it to be involved in that.” (Participant #4)
Positive Experiences and Impact Subtheme 1: Connecting Through a Shared Experience Subtheme 2: Feeling Validated and Empowered—Making Something Positive out of a Negative Experience**3**.Is it possible to put the two subthemes on separate lines?	“It was interesting being part of all of that and hearing everybody's input was important to me, and I learned a lot from that, [it] wasn't just about me, it was about everybody else's experiences as well.” (Participant #1) “But I also really enjoyed hearing the stories of the other participants and hearing that I wasn't alone and that the fear and the … the issues and the worries that I had were similar to what they experienced, and it just felt nice to know that that was a shared experience.” (Participant #3) “I felt represented and I need to speak to other patients that were part of this, and they said the same thing, like we feel represented, so therefore we know that you're going to be representing tons of people out there and in very close experience as to what we had.” (Participant #4) “Well, first of all, it validated my worries, but it also made me feel better in a way, made me feel better that there were other people out there like me. It wasn't just me. I wasn't alone. And they were doing all kinds of research on their own to find things that were helpful. And … they were sharing those things as well.” (Participant #1) “I found it validating for someone that was quite disabled when the project started.” (Participant #2) “So just making me feel useful and using the skills that I still have, using [not] just knowledge for my lived experience, but also just some of the other knowledge that I that I had from before.” (Participant #4)
Facilitators of Patient Partner Engagement	“It gave me quite a boost to be able to realize I do have skills of value. How it affected me as well is partly because of the way you in the project treated patient partners and we were treated with respect as having skills and information perhaps they didn't have.” (Participant #2) “I found the team super professional, super prepared, very clear. The instructions were clear. The mandate was clear. Our role was clear. I was happy to be consulted along the way with how things were progressing. Feedback I provided and that I saw other participants provided was incorporated. Clearly, that team was listening and I think the product that was developed is very high quality and professionally done, and kudos to the team [for doing] such an outstanding job.” (Participant #3) “Well, it was very positive. I felt very comfortable with everybody, including the other patients that were on. A lot of us have issues cognitively now or with fatigue and just not feeling well and just being able to participate. I think from the comfort of our home and everybody being so understanding that maybe when we're speaking or trying to share a thought, it might be a little bit hard for us due to our illness. It just made me feel very comfortable and I just I really appreciate the I think the way that everything was handled, actually, it makes me it makes me think that you guys are actually teaching what you're preaching or, for example, doing what you're what you're wanting to set out to do with others. So it just made me feel that everyone involved really cared. And that is such a breath of fresh air.” (Participant #4)
Barriers and Drawbacks to Patient Partner Engagement	“There was there was a couple of times where I wasn't feeling very well and really didn't want to participate in some of the Zoom calls.” (Participant #1) “I guess probably just the sort of the back-end piece, like a lot of time and work on your team and your end went into doing this with many steps and many layers and. I understand why that's necessary, and it produced a really good product, but I just wish we could have somehow made it happen a little faster.” (Participant #3) “I think, if anything, it's just my own health. I think I had to skip a meeting or so due to my own illness that I have now.” (Participant #4)

*Contributing and Giving Back.* When exploring reasons for participating in this project, PP described wanting to leverage their experiences with COVID-19 to contribute meaningfully to the medical research community, help others and themselves by gathering and sharing knowledge. PP wanted to make a difference by raising awareness regarding the need for improved patient experiences in the healthcare system during COVID-19.

*Positive Experiences and Impact.* PP highlighted that participation in this project resulted in positive experiences and impacts. Two subthemes were identified: connecting through a shared experience and feeling validated and empowered, which was explained as “making something positive of a negative experience.” PP described how they appreciated opportunities to connect with others in the group who shared similar experiences. While not all experiences were the same, PP were able to share strategies and support one another. PP reported negative interactions with the healthcare system as they sought a diagnosis early in the pandemic and felt hopeless and frustrated with the lack of empathy and compassion. However, through participation in this project, PP were able to turn their experiences into something meaningful and transformative, resulting in feelings of validation and empowerment.

*Facilitators, Drawbacks, and Barriers.* PP identified facilitators such as feeling heard, being valued, being treated with respect, feeling comfortable, strong project management, and a team that exemplified the project values. Specifically, PP highlighted that the research team fostered an environment that facilitated trust and respect, which enabled them to share their feelings and experiences openly and honestly. In contrast, PP commented on the length of time required to complete the project, highlighting differences between public and private sectors as well as their own health status as barriers. Several PP experienced post-COVID symptoms, which at times impeded their ability to engage or participate in the project.

## Discussion

Our findings suggest that PP were motivated to participate due to a desire to contribute, give back, and help themselves and others. These findings are congruent with patient engagement research where primary motivations to participate include improving the healthcare system, learning, and serving the community.^3^ Similarly, self-fulfillment, helping others, influencing healthcare, as well as affecting change were among the factors described as motivating patient engagement in the literature.^2^ In addition to their lived experiences, PP contributed knowledge and expertise to the project with their background in areas such as filmmaking, scientific writing, and education. This resulted in the development of a higher quality product that more accurately reflected PP experiences by harnessing skillsets outside of healthcare, resulting in more meaningful contributions.^9^

PP highlighted the value of connecting with others through a shared experience, knowing they were not alone, as well as learning from one another. Relationships with other patients have been reported to facilitate and enable the engagement process; some participants have also sought guidance and mentorship from other PP.^3^ Making meaningful connections and a sense of purpose have been identified as motivating engagement among PP.^2^ As such, this may be one way of promoting future patient engagement by leveraging the opportunity to connect with others through shared experiences. Both connecting with others and being able to give back contributed to feelings of validation and empowerment among PP. Findings are consistent with evidence suggesting patient participation results in feelings of competence and empowerment, increased feelings of self-worth, dignity, and overall satisfaction.^1-3,8^

Common facilitators that supported PP engagement included feeling heard, being valued, and treated with respect.^1,3,9,12^ Feeling comfortable with the team was articulated throughout the interviews, PP described an environment where they felt listened to and believed, enabling them to share their experiences and input. Findings reflect how the team lived our own values throughout the study to ensure a true authentic reciprocal experience for the PP as well as the research team. PP articulated that effective project management and participatory approaches facilitated engagement with clear delineation of roles, active engagement throughout the project and seeing PP input reflected in decisions.^1,3,5,9,12,13^ Project organization, leadership, and support for PP have been identified as contributing to positive, active engagement versus passive engagement.^1,3^

Although not raised within our study, compensation and appropriate resources may serve as a facilitator or if lacking, a barrier to engagement.^1,3,4,12,13^ PP were provided with a small token of appreciation for participation, with compensation limited by funder guidelines.^16-18^ Research teams should advocate for changes to these limitations given that PP contribute time, effort, and expertise that should be fairly compensated. Restrictions on funding guidelines are contradictory to the prioritization of equity, diversity, and inclusion principles across the research continuum. Further, receiving appropriate orientation and training has been identified as essential for supporting successful engagement.^1,3,5,9,12^ This was not addressed in PP interviews; however, orientation to the project and training about how best to share their stories at the outset was part of our PP onboarding process.

Barriers experienced by PP engaged in this project were not surprising. PP commented on the length of time required to complete the project, citing differences between public and private sector project expectations. Given that this project occurred during the pandemic, the process took longer than anticipated and was seen as a challenge to the knowledge mobilization goal. PP brought lived experience from the private sector, where projects often move more quickly than in academic research, highlighting the importance of clear communication and transparency related to project timelines and subsequent delays.^9,13^ PP health status also challenged active engagement,^8^ sometimes limiting participation in project meetings. This is an important consideration for future patient engagement, especially for PP experiencing health issues and/or chronic illness(es). Research teams should aim for flexibility as well as adaptability in scheduling, participation and contribution activities, and communication strategies.

## Limitations

While the project team aimed to recruit a diverse group of PP, those who applied to participate were similar in demographic characteristics.^2,3,19^ The research team realized early on the need to explore strategies to ensure broader representation in patient engagement.^4,9^ Conducting this research during the pandemic was another limitation, as most research was slowed or halted, and generally deemed non-essential.^19^ Recruitment was also strained as calls for participation occurred in the summer of 2020 (towards the end of the first wave), resulting in a smaller sample size than anticipated. Once recruited, our team was further limited to virtual engagement with PP, with most communication occurring through Zoom and email. Best practices in patient engagement support in-person interactions as they promote the development of rapport and allow the team to see PP as “real people.”^4^

## Conclusion

Engaging PP in the research process results in benefits for patients, healthcare providers, and the broader health system. This paper described PP experiences related to a project aimed at developing and pilot testing a VR video, highlighting benefits, facilitators, and barriers to engagement. As one of the participants so eloquently stated when reflecting on their overall experience: “it's a gift”—for both PP and the study team.
